# “Protective” Imazalil vs Its Negative Effects on Immune System Cells—Neutrophils

**DOI:** 10.3390/life16020365

**Published:** 2026-02-23

**Authors:** Wioletta Ratajczak-Wrona, Aleksandra Aniszewska, Agnieszka Iwaniuk, Marzena Garley, Sławomir Wołczyński, Dariusz Jan Skarżynski, Jolanta Wrobel, Agnieszka Zebrowska, Piotr Radziwon, Barbara Pucelik, Ewa Jabłońska

**Affiliations:** 1 Department of Immunology, Medical University of Bialystok, ul. Waszyngtona 15A, 15-269 Bialystok, Poland; 2 Department of Clinical Immunology, Medical University of Bialystok, Waszyngtona 17, 15-274 Białystok, Poland; 3 Department of Reproduction and Gynecological Endocrinology, Medical University of Bialystok, ul. M. Sklodowskiej-Curie 24a, 15-276 Bialystok, Poland; 4 Department of Immunology and Reproductive Pathology, Institute of Animal Reproduction and Food Research, Polish Academy of Sciences, ul. Trylinskiego 18, 10-683 Olsztyn, Poland; d.skarzynski@pan.olsztyn.pl; 5 Regional Centre for Transfusion Medicine, Marii Sklodowskiej-Curie 23, 15-275 Bialystok, Poland; 6 Lukasiewicz Research Network—Krakow Institute of Technology, ul. Zakopianska 73, 30-418 Krakow, Poland

**Keywords:** imazalil (IMZ), neutrophils, chemotaxis, phagocytosis, NO

## Abstract

Neutrophils are the most numerous population of peripheral blood leukocytes and play a key role in innate immunity, participating in antibacterial, antifungal, antiviral, and antitumor responses. Their activity can be modulated by endogenous and exogenous factors, including pesticides, among which fungicides such as the commonly used imazalil (IMZ) constitute a significant group. The objective of this study was to assess the effect of IMZ (at an environmental dose of 0.07 ng/mL, as well as 10- and 100-fold higher doses) on selected functions of neutrophils. This study demonstrated that neutrophils incubated with IMZ at a dose detectable in the serum, exhibited lower phagocytotic capacity. In addition, at a 10-fold higher dose, this compound reduced the chemotactic capacity of neutrophils and led to increased activity of NADPH oxidase in these cells. Furthermore, it was observed that at the highest concentration used in this study IMZ intensified the production of nitric oxide. The exposure of neutrophils—the first line of defense—to IMZ affected their locomotion and pathogen-eliminating function. Moreover, the response of neutrophils was not proportionate to the concentration of IMZ used in the study.

## 1. Introduction

Neutrophils constitute the largest fraction of circulating leukocytes and are the first line of defense. The main function of these cells is to eliminate pathogens (bacteria, fungi, virus-infected cells, tumor cells) through a series of nonspecific immune response mechanisms, including phagocytosis, degranulation, and formation of neutrophil extracellular traps (NETs) [1,2,3,4]. Neutrophils also participate in specific responses, for example, by secreting numerous cytokines and growth factors [5,6]. The activity of these cells can be affected by various substances of endogenous and exogenous origin, including widely used pesticides [7,8,9].

Currently, there are over 4000 substances and about 1000 preparations classified as pesticides. In the regulatory and scientific literature, including guidelines issued by international organizations such as the World Health Organization (WHO), the term, “pesticide” is defined as a plant-protecting product [10]. The global use of pesticides amounts to 2 million tons per year, and Europe accounts for nearly half of the global production [11]. In Poland, the sales of active substances included in pesticides amounted to over 23,000 tons in 2018 [12].

According to the World Health Organization, pesticides are substances that are potentially toxic to humans and other organisms. Therefore, it is important to follow manufacturer instructions and appropriate precautions during the use, storage, and disposal of such substances [13,14,15].

Pesticides, including fungicides, are divided into numerous groups due to their high diversity and criteria referring to their mechanisms of action or use [13,16]. Due to its high antifungal efficacy, the fungicide imazalil (IMZ) has been widely used since the 1970s [17,18,19].

IMZ 1-[2-(2,4-dichlorophenyl)-2-(2-propenyloxy)-ethyl]-1H-imidazole is a synthetic pesticide, which belongs to the group of imidazoles [20,21]. It is a chiral compound, featuring an asymmetric carbon in the C7 position due to which it selectively binds with human albumin [22]. IMZ has lipophilic properties, moderately dissolves in water, and is characterized by a high n-octanol/water partition coefficient. Under aerobic conditions, this compound shows very slow degradation in soil, but under the influence of sunlight, it disintegrates rapidly [23]. IMZ is also classified as a compound with potential endocrine-disrupting chemicals—EDCs [24,25].

Throughout the European Union, IMZ is used in agriculture (commercial names: Fungaflor and Fungazil) to protect fruits, vegetables, and wheat from molds. It is also applied in veterinary medicine (commercial name: Enilkonazol) to treat dermal mycosis in dogs and in smoke generators in chicken hatcheries [18,21,22,23]. The maximum dose of IMZ used after citrus harvest is 5 mg/kg, whereas in the case of bananas, the maximum dose is 2 mg/kg. Although IMZ is applied on inedible parts of fruits and vegetables, consumers are exposed to this compound as it can penetrate the flesh of fruits and vegetables through their skin. In agricultural areas, its concentration in water is estimated at approximately 1 mg/L [26,27,28,29]. For this reason, IMZ is considered a widespread food and environmental pollutant. In human serum, IMZ is detectable at a concentration of 0.07 ng/mL [30]. Data reveal that IMZ administered to animals at a high therapeutic dose of 200 mg is quickly eliminated from the organism and is not subject to bioaccumulation. The half-life of this compound in the serum is approximately 2 h [27].

The main target of IMZ is P450 cytochrome aromatase. Disturbances in the function of this enzyme block cell wall synthesis in fungal cells. On the other hand, in animals, IMZ affects the de novo synthesis of cholesterol, the basic substrate for the production of steroid hormones, among other substances. In addition, by acting as an androgenic receptor antagonist, IMZ blocks the conversion of androgen to estrogens [31,32].

Although IMZ was characterized as a substance causing moderate toxicity, testing on animal models has shown a range of negative effects including teratogenicity, genotoxicity, hepatotoxicity, and disturbances in the hormonal and nervous system, and its potential to cause intestinal dysbiosis [26,28,33,34].

In recent years, IMZ has been considered an immunotoxin, that is, a chemical that affects the human organism and causes disturbance or death of cells of the immune system.

Studies based on functional methods play a significant role in toxicological research on the assessment of the level of immunotoxicity of a given compound. Such methods allow assessing if—and to what degree—an immunotoxin may affect the functional status of diversified cells constituting the immune system. Based on the above prerequisites, we undertook a study to assess the effect of IMZ on the basic functions of human neutrophils, such as chemotaxis, phagocytosis, oxidative burst, production of nitric oxide (NO). Based on the available literature [30], the study utilized IMZ at a concentration of 0.07 ng/mL. However, considering the fact that some xenobiotics exhibit a nonmonotonic relationship between dose and effect, we examined 10- and 100-fold higher concentrations than the concentration detected by Chang et al. [30] in the human blood serum.

## 2. Methodology

### 2.1. Materials

The study material was venous blood collected from the basilic vein of 15 voluntary blood donors (aged 23–32 years) from the Regional Centre for Transfusion Medicine, Białystok. The donors were healthy, nonsmoking men, and did not consume alcohol for at least 48 h before the blood collection. The study group consisted only of men, which allowed us to eliminate the dependence of the obtained test results on hormonal fluctuations related to the menstrual cycle. Every donor was informed of the study objective and provided written consent for the use of the blood samples for research purposes. The blood samples were collected in a test tube with heparin (10 U/mL; Heparin, Polfa, Łódź, Poland).

### 2.2. Neutrophil Isolation

**Figure 1:**
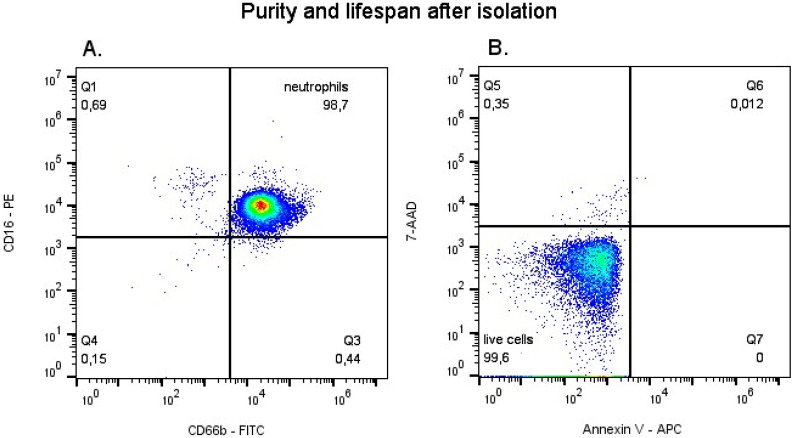
Purity and lifespan after isolation assessed by flow cytometry technique. (**A**) Neutrophil purity was assessed directly after the isolation, by marking a population of cells with CD16+ CD66b+. (**B**) Cell viability assessment was performed directly after isolation by staining with Annexin V and 7-AAD.

Neutrophils were isolated from whole blood by applying centrifugation in the gradient of density Polymorphprep™ (Axis-Shield, Oslo, Norway). This gradient resulted in two cell fractions—PMNs (containing 91% neutrophils) and peripheral blood mononuclear cells. To obtain pure neutrophil fraction (99.9%), the PMN suspension was rinsed with PBS without MgCl^2+^ and CaCl^2+^ ions (Gibco, Thermo Fisher Scientific, Waltham, MA, USA) and positive magnetic separation Midi-MACS (Miltenyi Biotec, Bergisch Gladbach, Germany) was used with monoclonal antibodies against the surface CD16 antigen [35,36]. The purity of the obtained cellular fraction was assessed by flow cytometry. To this end, the cells were stained with anti-CD16 antibodies (BD Pharmingen, San Jose, CA, USA) and anti-CD66b (BD Pharmingen, San Jose, CA, USA), and after incubation, cytometric analysis was performed using the Accuri C6 Plus cytometer (BD Biosciences, San Jose, CA, USA) neutrophils constituted 99% of the isolated cell fraction (Figure 1A). Cell viability was also assessed by cytometry after incubation with Annexin V (BD Biosciences, San Diego, CA, USA) and 7AAD (BD Biosciences, San Jose, CA, USA). Data analysis was performed using the FlowJo program (FlowJo™ Software, ver. 10.9, Becton, Dickinson and Company, Ashland, OR, USA); it was maintained at the level of 98% (Figure 1B).

### 2.3. Neutrophil Chemotaxis

**Figure 2:**
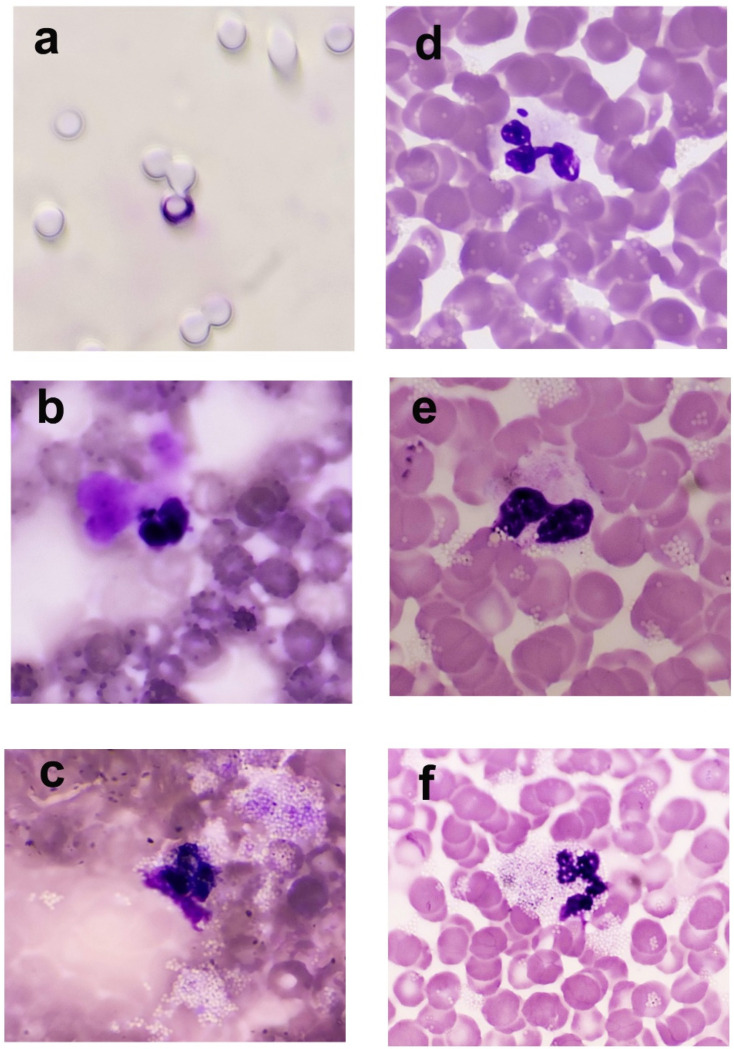
Representative photographs of neutrophils. Slides were stained using May–Grünwald and Giemsa stains. Cells were counted manually in a light microscope. Magnification × 100. (**a**) Neutrophil ability of chemotaxis was evaluated using the Boyden chamber; cell staying in the membrane pore. (**b**,**c**) Activity of NADPH oxidase in neutrophils was evaluated using the NBT test; (**b**) neutrophil with formazan crystal in the spontaneous test. (**c**) Neutrophil with latex beads and formazan crystal in the cytoplasm in the stimulated test. (**d**–**f**) Neutrophil ability of phagocytosis was assessed using Park’s test with latex beads: (**d**) neutrophil in the first group with up to 10 latex beads in the cytoplasm; (**e**) neutrophil in the second group with 11–30 latex beads in the cytoplasm; (**f**) neutrophil in the third group with more than 30 latex beads in the cytoplasm.

The capacity of neutrophils for targeted movement toward increasing concentration of the chemotactic factor (chemotaxis) was assessed by quantitative method using the Boyden chamber (Neuro Probe, Inc. Gaithersburg, MD, USA) [37]. PMNs obtained in density gradient centrifugation were preincubated for 60 min in the presence of IMZ (0.07, 0.7, and 7 ng/mL) (Sigma-Aldrich, Merck, St. Louis, MO, USA) or without the investigated compound. In the Boyden chamber comprising two parts, and divided by a Millipore filter with a pore diameter of 5 µm (Merck Millipore, Billerica, MA, USA), one chamber was filled with a solution of the chemotactic factor—N-formyl-Met-Leu-Phe (40 ng/mL) (Merck Millipore, Burlington, MA, USA), while the second chamber (above the Millipore filter) was filled with a suspension of 50,000 neutrophils that were preincubated with or without IMZ. The filled Boyden chamber was incubated in an incubator with 5% CO_2_ flow at 37 °C for 1 h. Then, the filter was placed on a slide; once dried, it was stained with the May–Grünwald–Giemsa method. The preparation was assessed under a light microscope (immersion, 100× magnification), counting all neutrophils present above and inside the filter. To calculate the percentage of neutrophils capable of chemotaxis, a mathematical formula was used, according to which 50,000 cells constituted 100% (Figure 2).

### 2.4. Neutrophil Phagocytosis

The percentage of neutrophils capable of phagocytosis and the efficiency of this process, expressed as the Social Cohesion and Reconciliation (SCORE) index, were evaluated using the Park method with latex particles [38]. To obtain a leukocyte buffy coat, the venous blood collected on heparin was centrifuged for 5 min (at 2000 rpm). The isolated cellular fraction was incubated for 60 min in an incubator with 5% CO_2_ flow (Nuarie™, Plymouth, MN, USA) at 37 °C without or with IMZ (0.07, 0.7, and 7 ng/mL). Subsequently, latex (Latex beads, polystyrene, 0.8 μm mean particle size, Merck Millipore, Burlington, MA, USA) was added to the suspension. The cell suspension was incubated first for 30 min in an incubator with 5% CO_2_ flow at 37 °C, and then at room temperature for another 30 min. Smears were prepared and stained using May–Grünwald–Giemsa reagents (Stamar, Dąbrowa Górnicza, Poland). The preparations were assessed under a light microscope (immersion, 100× magnification). During this process, 100 neutrophils were counted, differentiating them based on the absorption of latex grains as follows: Class 0—neutrophils without latex in the cytoplasm; Class I—neutrophils containing 1–10 latex grains in the cytoplasm; Class II—neutrophils containing 11–30 latex grains in the cytoplasm; Class III—neutrophils containing over 30 latex grains in the cytoplasm. The percentage of cells capable of phagocytosis (quantitative assessment of phagocytosis) was calculated using the formula: (100 − number of cells in Class 0) × 100%; SCORE index (qualitative assessment of phagocytosis) was calculated using the formula: (number of cells in Class 0 × 0) + (number of cells in Class 1 × 1) + (number of cells in Class 2 × 2) + (number of cells in Class 3 × 3) (Figure 2).

### 2.5. NADPH Oxidase Activity in Neutrophils

The activity of NADPH (enzyme catalyzing the formation of superoxide anion radicals) was assessed in the IMZ-exposed neutrophils by nitroblue tetrazolium (NBT) reduction test as per Park [38]. The test is based on the absorption of the NBT stain by neutrophils. Under the influence of NADPH oxidase, the stain is reduced to violet, insoluble deposits of formazan in the cytoplasm of neutrophils. The blood collected from donors was incubated for 60 min in an incubator with 5% CO_2_ flow (Nuarie™, Plymouth, MN, USA) at 37 °C with or without IMZ (0.07, 0.7, and 7 ng/mL). In the next step, NBT (Sigma-Aldrich, Merck, St. Louis, MO, USA). Two tests were simultaneously conducted: NBT spontaneous (containing the NBT reagent) and NBT stimulated (containing NBT reagent and latex (Latex beads, polystyrene, 0.8 μm mean particle size, Merck Millipore, Burlington, MA), which further stimulates the absorption process). The prepared test tubes were incubated for 15 min in an incubator with 5% CO_2_ flow (Nuarie™, Plymouth, MN, USA) at 37 °C, and for another 15 min at room temperature. Subsequently, smears were prepared and stained using the May–Grünwald–Giemsa method. The preparations were assessed under a light microscope (immersion, 100× magnification). In the spontaneous NBT test, 100 neutrophils were divided into those containing no formazan deposits in the cytoplasm and those with formazan deposits in the cytoplasm. The test result consists of the percentage of neutrophils containing formazan deposits in the cytoplasm. In the stimulated NBT test, 100 neutrophils were counted based on the presence of formazan deposits and latex grains in the cytoplasm. The test result consists of the percentage of neutrophils containing both formazan deposits and latex grains in the cytoplasm (Figure 2).

### 2.6. Incubation of Neutrophils

For incubation with IMZ, the isolated neutrophils (isolated in centrifugation in density gradient and positive magnetic separation with microbeads labeled with monoclonal anti-CD16 antibody (Miltenyi Biotec, Bergisch Gladbach, Germany) were first suspended in a culture medium, containing Rosewell Park Memorial Institute 1640 (Gibco; Thermo Fisher Scientific, Waltham, MA, USA), antibiotics: 100 U penicillin/mL and 50 ng streptomycin/mL(Gibco; Thermo Fisher Scientific, Waltham, MA, USA), and serum (4%); newborn calf serum (NCBS) (Gibco, Thermo Fisher Scientific, Waltham, MA, USA) and applied to the wells of a 96-well Falcon-type plate. The cells were incubated for 2 h at 37 °C with 5% CO_2_ (Nuarie™, Plymouth, MN, USA) without or in the presence of IMZ (0.07, 0.7, and 7 ng/mL) [27]. After incubation, the culture plate was centrifuged, and the resulting supernatant was used to assess the production of NO.

Cell viability was assessed after incubation by calculating the percentage of live cells using the Accuri C6 Plus cytometer (BD Biosciences, San Jose, CA, USA). To this end, the cells were stained with Annexin V and 7-AAD (BD Biosciences, San Jose, CA, USA), incubated for 20 min, and then subject to cytometric analysis, using the FlowJo program (FlowJo™ Software, ver. 10.9, Becton, Dickinson and Company, Ashland, OR, USA)

### 2.7. Nitric Oxide Production

NO production was assessed using the intermediate method based on the Griess reaction [39]. This method comprises two stages and enables the detection of released nitrates (III) and (V). The total NO concentration is equal to the sum of the concentration of nitrates (III) and (V). In the first stage involving a 30 min incubation with cadmium (Sigma-Aldrich, Merck, St. Louis, MO, USA), nitrates (V) were reduced to nitrates (III). Subsequently, the supernatant was incubated for 30 min with Griess reagent (Sigma-Aldrich, Merck, St. Louis, MO, USA) at room temperature. The absorbance was measured at a wavelength of λ = 540 nm on a spectrophotometric reader (Varioskan™ LUX Multimode Microplate Reader, Thermo Fisher Scientific, Waltham, MA, USA) The concentration of NO products was expressed in µM (10^6^ cells in 270 mL supernatant).

### 2.8. Statistics

Data were analyzed using Statsoft Statistica, version 13.3 (StatSoft, Inc., Tulsa, OK, USA). The statistical significance of any difference in each parameter among the groups was evaluated by one-way analysis of variance, using Tukey’s multiple comparisons test as a post hoc test. The results are expressed as median and interquartile range (IQR). Boxes represent IQR, the horizontal line indicates the median, whiskers show the range, and dots represent individual values. Significance was defined as *p* < 0.05.

## 3. Results

### 3.1. Neutrophil Chemotaxis

The chemotaxis assay quantifies the ability of leukocytes to undergo directional migration in response to chemoattractant concentration gradient. This study aimed to determine the effect of IMZ exposure on neutrophil chemotactic function.

**Figure 3:**
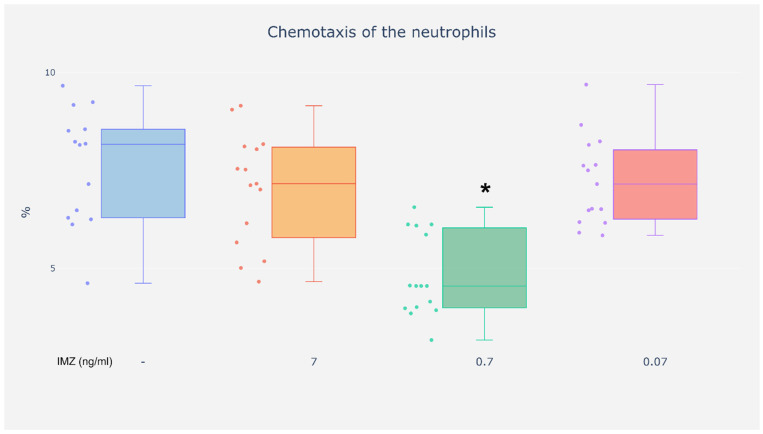
Chemotaxis of the neutrophils. %—Percentage of neutrophils capable of chemotaxis. Value significantly different between: * cells without and with IMZ (0.7 ng/mL) (*p* < 0.05).

No differences in chemotaxis capacity were observed in the neutrophils exposed to IMZ at the concentrations of 0.07 and 7 ng/mL as compared with unexposed cells (Figure 3). However, exposure to IMZ at the concentration of 0.7 ng/mL caused a reduction in chemotaxis capacity in neutrophils as compared with cells incubated without the investigated compound (Figure 3).

The results suggest that IMZ may modulate neutrophil chemotaxis in a condition-dependent manner.

### 3.2. Neutrophil Phagocytosis

The latex phagocytosis assay evaluates the capacity of neutrophils to internalize particulate targets, representing the initial step of the phagocytic process, as quantified by the number of ingested latex particles. The assay determines both the proportion of phagocytosing cells and a phagocytic efficiency index. This study aimed to evaluate neutrophil phagocytic capacity following exposure to IMZ.

**Figure 4:**
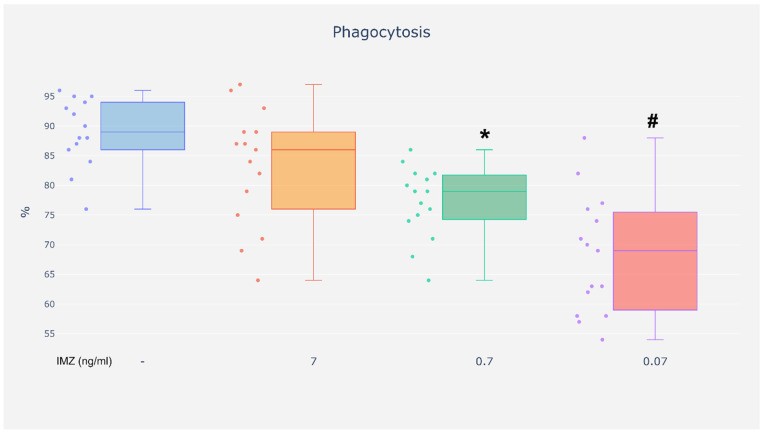
Phagocytosis–the percentage of phagocytic cells. %—the percentage of neutrophils capable of phagocytosis (quantitative analysis). Value significantly different between: * cells without and with IMZ (0.7 ng/mL) (*p* < 0.05); #—cells without and with IMZ (0.07 ng/mL) (*p* < 0.05).

Exposure to IMZ at the concentrations of 0.07 and 0.7 ng/mL resulted in a reduced percentage of neutrophils capable of phagocytosis as compared with the cells incubated without the investigated compound (Figure 4). However, there were no changes in the percentage of neutrophils capable of phagocytosis after exposure to IMZ at the concentration of 7 ng/mL compared with unexposed cells (Figure 4).

**Figure 5:**
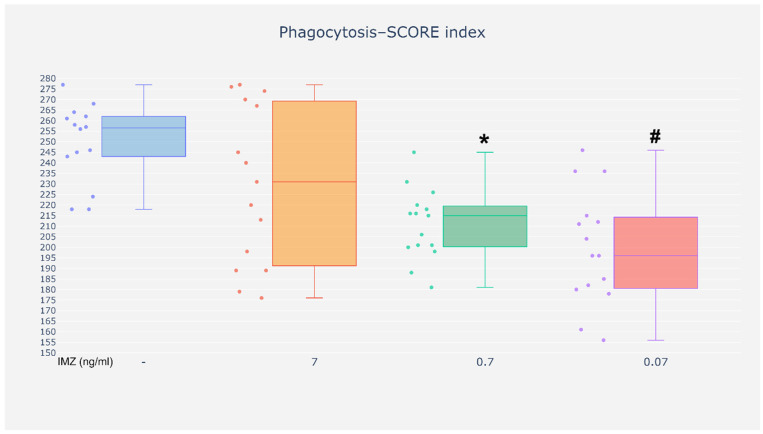
Phagocytosis–SCORE index. SCORE index—efficiency of the phagocytosis process (quantitative assessment using the Social Cohesion and Reconciliation—SCORE index). Value significantly different between: * cells without and with IMZ (0.7 ng/mL) (*p* < 0.05); #—cells without and with IMZ (0.07 ng/mL) (*p* < 0.05).

Analysis of the phagocytosis test results based on the SCORE index revealed reduced phagocytic activity in neutrophils exposed to IMZ at the concentrations of 0.07 and 0.7 ng/mL as compared with the cells incubated without the investigated compound. However, incubation with IMZ at the concentration of 7 ng/mL did not affect the phagocytic activity of neutrophils (Figure 5).

Exposure to IMZ appeared to modulate neutrophil phagocytic function under specific conditions.

### 3.3. NADPH Oxidase Activity in Neutrophils

The NBT assay evaluates the capacity of neutrophils to perform intracellular killing via reactive oxygen species, based on the reduction of NBT by NADPH oxidase, the enzyme responsible for initiating the respiratory burst. The spontaneous assay reflects the activation state of neutrophils at the time of blood collection, whereas the latex-stimulated assay assesses whether particle uptake effectively triggers NADPH oxidase activation. The study aimed to assess the potential impact of IMZ on NADPH oxidase activity.

**Figure 6:**
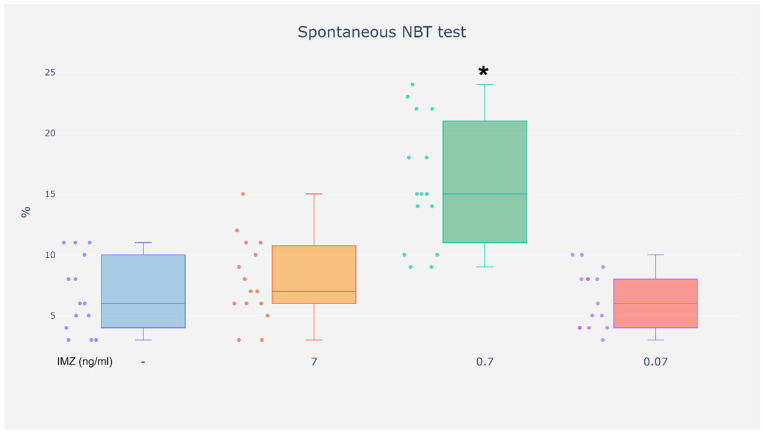
Spontaneous NBT test. %—Results are expressed as the percentage of neutrophils containing formazan deposits in the cytoplasm. * cells without and with IMZ (0.7 ng/mL) (*p* < 0.05).

The spontaneous NBT test results indicated increased activity of NADPH oxidase in the neutrophils exposed to IMZ at the concentration of 0.7 ng/mL as compared with cells incubated without the tested compound. Meanwhile, exposure of neutrophils to IMZ at the concentrations of 0.07 and 7 ng/mL did not cause any changes in the percentage of NBT-positive cells (Figure 6).

**Figure 7:**
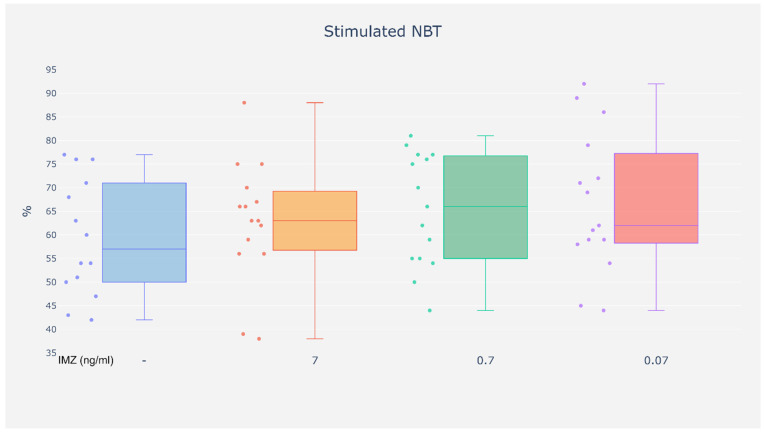
Stimulated NBT. %—Results are expressed as the percentage of neutrophils containing both formazan deposits and latex grains in the cytoplasm.

However, in the stimulated NBT test, no increase in the percentage of NBT-positive cells was observed in the presence of IMZ at any of the tested concentrations (0.07, 0.7, and 7 ng/mL) as compared with unexposed cells (Figure 7).

The results suggest that IMZ may influence neutrophil NADPH oxidase activity in a condition-dependent manner.

### 3.4. Viability of Neutrophils After Incubation with IMZ

The study aimed to evaluate the potential impact of IMZ on neutrophil viability.

**Figure 8:**
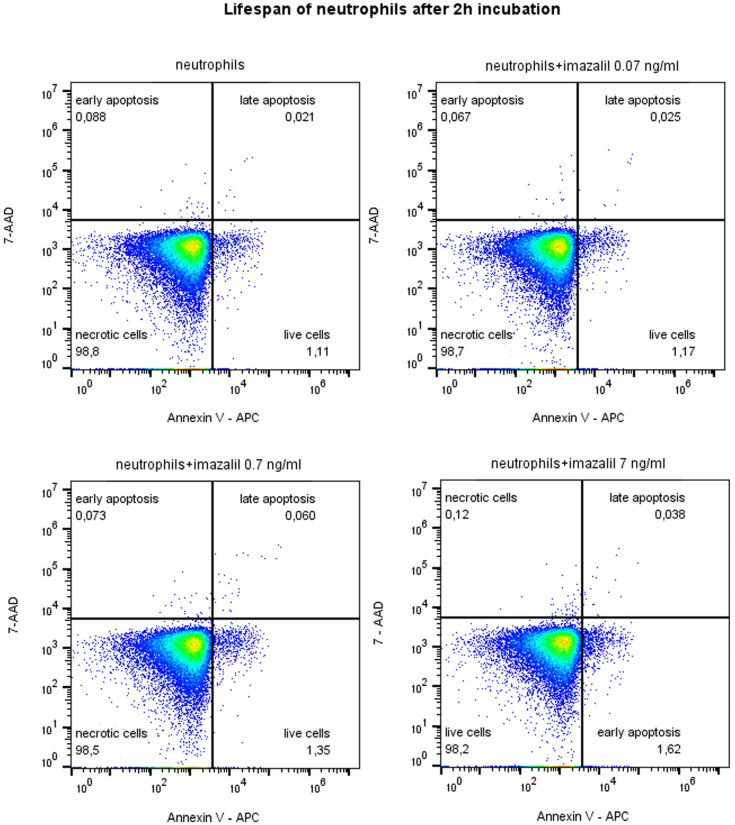
Lifespan of neutrophils after 2h incubation with imazalil (0.07 ng/mL, 0.7 ng/mL, 7 ng/mL) using flow cytometry method. Assessment of the percentage of live neutrophils (7-AAD-; Annexin V-).

A 2 h exposure of neutrophils to IMZ (0.07, 0.7, and 7 ng/mL) did not affect the viability of tested cells. The percentage of live neutrophils remained at the level of approximately 98% (Figure 8).

Exposure to IMZ did not appear to affect neutrophil viability under the tested conditions.

### 3.5. Neutrophil NO Generation

The aim of this experiment was to assess whether IMZ modulates nitric oxide release by neutrophils.

**Figure 9:**
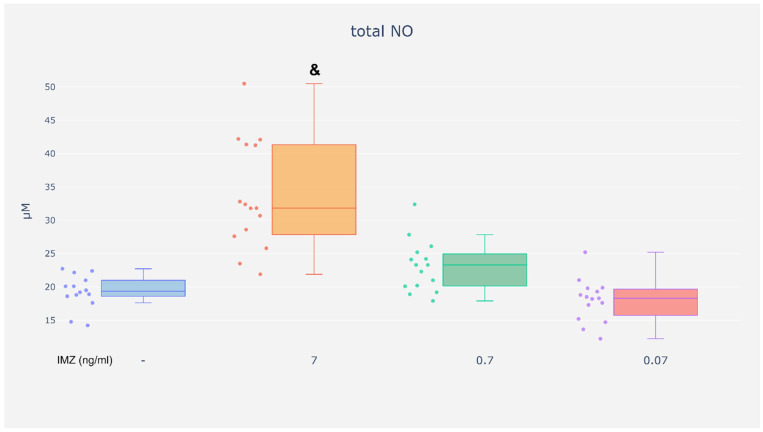
Concentrations of total NO in the neutrophils supernatants. Neutrophils were treated without or with IMZ (0.07 ng/mL, 0.7 ng/mL or 7 ng/mL) and then the supernatants were subjected to nitrite assay. Value significantly different between: &—cells without and with IMZ (7 ng/mL) (*p* < 0.05).

No changes in NO generation could be found in neutrophils exposed to IMZ at the concentration of 0.07 or 0.7 ng/mL (Figure 9). However, exposure to IMZ at the concentration of 7 ng/mL resulted in increased release of NO by neutrophils as compared with unexposed cells (Figure 9).

The results suggest that IMZ may influence neutrophil nitric oxide production under specific conditions.

## 4. Discussion

The findings presented here demonstrate for the first time the diverse, dose-dependent effects of IMZ on human neutrophils.

In the study, neutrophils incubated with IMZ at a dose that is detectable in human serum showed reduced capability for phagocytosis. In addition, at a 10-fold higher dose, IMZ reduced the capability of these cells for both chemotaxis and phagocytosis. Therefore, it can be concluded that IMZ has an inhibiting effect on the functions of neutrophils.

However, the assessment of NADPH oxidase activity in the presence of IMZ at the concentration of 0.7 ng/mL showed a different—stimulating—effect of IMZ on neutrophils. Balistrieri A et al. observed increased NADPH oxidase activity due to the action of bisphenol A, a compound classified among endocrine-disrupting compounds (EDCs), in their study [40]. However, the concentration of bisphenol A used by those researchers was almost threefold higher than the concentration of IMZ producing the same effect. Increased activity of NADPH oxidase intensifies the production of reactive oxygen species (ROS) which may disturb the cellular pathways sensitive to redox reactions. By oxidizing BH4 (tetrahydrobiopterin cofactor), ROS reduce the production of NO, a factor responsible for the formation of smooth muscles in blood vessels and preventing the aggregation of blood platelets on the vessel surface. Excessive amount of ROS also contributes to, among others, increased expression of adhesion particles and endothelial permeability, thereby causing the accumulation of leukocytes and LDL molecules in the wall of blood vessels. The above reactions may consequently lead to changes that predispose to the development of atherosclerosis. It has also been demonstrated that excessive concentration of ROS increases the possibility of cancer development because ROS cause DNA damage and elevate the expression of certain proto-oncogenes [41,42,43,44,45,46,47,48].

In the case of NO production by neutrophils, a stimulating effect of IMZ was observed in our experiments, but only at the highest concentration used.

It can therefore be concluded that the observed differences in neutrophil responses to imazalil may partially result from the diversity of mechanisms underlying intra- and extracellular signaling involved in phagocytosis, chemotaxis, activation of NADPH oxidase, and nitric oxide synthesis. The interdependence of signaling pathways constitutes an integrated network of intracellular information flow, and activation of any component of this network affects the state of the others.

The study showed that IMZ caused significant changes in basic neutrophil functions, even at doses that can be found in the serum in physiological state. Interestingly, the results reveal that the effect of IMZ on neutrophils was not proportionate to the exposure dose. In the majority of experiments, the greatest change in neutrophil function was observed with the intermediate dose (0.7 ng/mL). Research conducted with EDCs is often combined with nonmonotonic dose–response (NMDR) relationships. The effect of NMDR is characterized by the most intensive response to the tested compound after its application at intermediate doses. At low concentrations, imazalil can perturb steroidogenic enzymes and hormone secretion, while higher doses trigger cytotoxicity or receptor saturation that masks these signaling effects—creating U- or inverted-U curves. There are no linear relationships over the range from low to high doses [49,50].

People are constantly exposed to EDCs. Rarely do any of them act alone; rather, their effects combine, and depending on their mechanisms of action, the effects can be cumulative and act additively or antagonistically. A study by Orton et al. shows the cumulative effect of 30 compounds, including 13 pesticides, including imazalil. In the study, striking low-dose combination effects of 30 mixtures produced a 100% inhibition of the agonistic effects [51].

According to the scheme created by Lagarde et al., the literature data concerning the mechanism of action of IMZ on neutrophils is insufficient to unambiguously determine the effect of NMDR [49]. However, considering the potential NMDR effect of IMZ, the negative impact of this fungicide on human organisms will not be proportionate to the level of concentrations applied. Thus, the standard toxicological approach used for assessing the harmfulness of the test substance—which is based on the relationship: the higher the dose, the worse the effects on the organism—may not be applicable for IMZ. Our team is the first to perform a preliminary assessment of the effect of IMZ on the defensive function of neutrophils. Thus, the present study should be treated as a pilot study. To assess the actual hazard posed by IMZ, studies should be conducted on a broader range of exposure doses, paying special attention to even lower concentrations of the fungicide. Moreover, further research is needed on a larger and more diverse group of subjects in terms of age and sex.

Conducting in vitro studies on a compound, while modeling the action of the endocrine system, it is difficult to clearly define the mechanism of the reaction induced by the xenobiotic, due to the complexity of the processes controlled by hormones. Therefore, in vivo studies should be conducted for such compounds, especially since recent research has demonstrated the increasingly broad and harmful effects of imazalil. Luis A. Valdivia-Chávez et al. reported that imazalil induces neurotoxic effects in rats by reducing cholinesterase activity in the serum and hippocampus and by impairing learning and memory functions [52]. It is also important to emphasize that the effects of imazalil exposure may affect not only directly exposed individuals but also subsequent generations. Studies by Cuiyuan Jin and colleagues demonstrated that exposure to imazalil during pregnancy led to disturbances in glycolipid metabolism and abnormal m^6^A RNA methylation in the livers of both mothers and their offspring in mice [53].

Moreover, a human organism is chronically exposed to numerous substances present in the environment or food. Thus, it should be taken into account that the effect of a mixture of toxic substances may be different and more unforeseeable than that of single xenobiotics [21].

## 5. Conclusions

In summary, the results of this study showed that IMZ affects the basic functions of neutrophils, which may lead to immunological disturbances. However, further research is needed to assess the mechanism of action of this compound on neutrophils, as well as its potential and unfavorable effects on the human organism.

## Data Availability

The datasets used and/or analyzed during the current study are available from the corresponding author on reasonable request.
